# Trueness of the Inner Surface of Monolithic Crowns Fabricated by Milling of a Fully Sintered (Y, Nb)-TZP Block in Chairside CAD–CAM System for Single-visit Dentistry

**DOI:** 10.3390/ma12193253

**Published:** 2019-10-05

**Authors:** Jun-Ho Cho, Hyung-In Yoon, Jung-Suk Han, Dae-Joon Kim

**Affiliations:** 1 Department of Prosthodontics, School of Dentistry and Dental Research Institute, Seoul National University, Seoul 03080, Korea; riddle1208@snu.ac.kr (J.-H.C.); proshan@snu.ac.kr (J.-S.H.); 2 Department of Dentistry, School of Dentistry and Dental Research Institute, Seoul National University, Seoul 03080, Korea; djkim2014@gmail.com

**Keywords:** CAD–CAM system, fabrication time, fully contoured monolithic crowns, dental zirconia blocks, fixed prosthodontics

## Abstract

A single-visit zirconia restoration can be easily achieved if direct milling of a fully sintered zirconia block can be performed without much effort. However, no studies have yet been reported regarding the evaluation of the trueness of crown fabricated from chairside-milling of a fully sintered zirconia block in the chairside computer-aided design and computer-aided manufacturing (CAD–CAM) system for single-visit dentistry. This in vitro study aimed to evaluate the trueness of crowns fabricated by milling a fully sintered zirconia block in the chairside CAD–CAM system and investigate the clinical implications for single-visit chairside restoration. Crowns were fabricated either by chairside-milling a fully sintered block of niobium oxide containing yttria-stabilized tetragonal zirconia polycrystals ((Y, Nb)-TZP) without the sintering process (n = 12) in a chairside single-visit dentistry system (Chairside group) or by laboratory-milling a partially sintered 3 mol% block of yttria-stabilized tetragonal zirconia polycrystals (3Y-TZP) followed by the sintering process (n = 12) in a conventional laboratory system (Labside group). Crown fabrication time, milling tool diameter and the trueness of each crown were evaluated. All trueness values of both groups were within the clinically acceptable range, although a significant difference between the Chairside (43.0 ± 3.67 μm) and Labside groups (37.4 ± 2.41 μm) was observed (P < 0.05). Mean fabrication time was 0.52 h and 1.42 h for Chairside and Labside groups, respectively. A decrease in the tool diameter was observed for the Chairside group.

## 1. Introduction

Along with the development of computer-aided design and computer-aided manufacturing (CAD–CAM) technology, the manufacturing process for dental prosthesis can be divided into either the chairside or the laboratory system. The chairside system allows clinicians to design and manufacture the dental prosthesis in their own dental clinics, which enables single-visit restorative treatment [1]. With the chairside CAD–CAM system, various ceramic materials have become candidates for single-visit restorations. However, a partially sintered monolithic zirconia cannot be used for a single-visit treatment because of the time-consuming fabrication, as post-milling sintering can take up to 12 h [2,3,4]. Recently, a speed (60 to 120 minutes) or high-speed (10 minutes) sintering process for the partially sintered zirconia has been developed, which greatly reduces the total fabrication time [2,3]. However, the mechanical or optical properties of monolithic zirconia fabricated with speed or high-speed sintering are not clearly known and are thus of clinical concern [2,3]. Therefore, in most cases, partially sintered zirconia blocks are used in laboratory CAD–CAM systems.

Zirconia ceramic is currently a widely used restorative material for fixed dental prosthesis due to its esthetic, biocompatible, and mechanical properties [5]. There are two types of dental zirconia blocks designed for CAD–CAM; one is a fully sintered (or post-sintered) block, and the other is a partially sintered (or pre-sintered) block. The fully sintered zirconia block can be used in milling at a 1:1 ratio, which needs no further sintering process. However, it requires a robust milling system with a high level of accuracy, especially in thin areas [6,7]. On the other hand, milling with a partially sintered zirconia block is cost-effective and easy to perform, but should be milled to a 25% larger size to compensate for the sintering shrinkage [8]. Even though the sintering process takes long time, the sintering process, which is unnecessary for a fully sintered block, has the potential to act as a type of “regeneration firing”, which induces self-healing of surface flaws caused during the milling process or reestablishment of the tetragonal phase [9,10,11]. It is generally accepted that the zirconia becomes more stable and reliable with favorable surface properties after self-healing, even though uncertainty about strength exists [9,12]. A fully sintered zirconia milling has slightly better marginal and internal fit than a partially sintered milling [6]. Since an optimal milling system for a dense, fully sintered zirconia block has not yet been suggested, the minor superiority of fully sintered zirconia milling with respect to the marginal and internal fit of the crown may not be comparable to the cost-effectiveness of a partially sintered zirconia milling [6].

Trueness represents the closeness of agreement between the measurement value and the true value [5,13,14]. Trueness can be evaluated using an optical scanner and 3-dimensional (3D) inspection software, allowing for superimposition analysis of measured data and reference data; this method is nondestructive and represents its result both numerically and visually [5,15]. This substitutes the conventional methods for measuring marginal and internal fit, such as the silicon replica technique or cross-section technique [5,16]. The root mean square (RMS) value is often used to express the trueness, with a low RMS value representing good trueness [17]. Peters et al. proposed that an RMS value of less than 10 μm is considered an excellent fit, whereas more than 50 μm is a poor fit [18]. However, there is no consensus or guideline for interpreting RMS value for a good or bad fit. Three- or four-axis milling machines produce a trueness of 61 ±22 μm for inner surfaces and 55 ±18 μm for occlusal surfaces [13,19], but 5-axis machines can achieve a more accurate prosthesis with a trueness of 41 ±15 μm for inner surfaces and 55 ±18 μm for occlusal surfaces [13,19]. Since monolithic crowns milled with a partially sintered zirconia block can shrink after sintering, both the machining accuracy and non-uniformity of sintering shrinkage can affect the trueness of definitive crowns [8,20].

The milling tools (or burs) are normally worn out during the milling process of dental zirconia crowns. Thus, it is necessary to evaluate and project the lifespan of a milling tool to replace it before it begins to produce low-quality restoration or damaging the milling equipment [21]. The life of the milling tools is expressed in the number of restorations manufactured, but the ability to endure machining while preserving an adequate level of accuracy and efficiency is not usually well-documented [21]. It is known that tool wear is affected by the type of materials to be milled [21,22], with harder materials leading to greater tool wear [22]. Since a fully sintered zirconia block is very hard and tough, milling tool wear should be considered. Tool wear can be reduced by optimizing milling parameters such as feed rate, cutting depth, cutting speed, and milling path [22]. Although there are no established criteria for the assessment of tool wear, a possible evaluation method is to assess the loss of diamond grit on the tool surface, change in diamond grit density, loss of tool mass, or change in the surface roughness of the tool’s surface [21,22].

This in vitro study aimed to investigate the clinical applicability of a chairside CAD–CAM system by milling a novel, fully sintered (Y, Nb)-TZP block as a single-visit full-contour monolithic crown restoration system. In a chairside CAD–CAM system, a fully sintered (Y, Nb)-TZP block was milled with a numerically controlled protocol and a novel diamond-milling tool. We compared the trueness of inner surfaces of full-contour monolithic zirconia crowns fabricated in a chairside CAD–CAM system and laboratory CAD–CAM system. The null hypothesis was that no difference would be found in the trueness of a full-contour monolithic crown milled between two groups. Crown fabrication time for the two groups was also compared, and the wear of the milling tool was examined on the basis of the measurement of the bur diameter as the milling cycles proceeded.

## 2. Materials and Methods 

### 2.1. Working Cast Preparation

A female patient who needed a full-contour crown for a maxillary right second molar had received tooth preparation treatment for restoration with monolithic zirconia. No patient-related clinical records were used in this study. The preparation was performed to provide 1.5 mm occlusal reduction, 1–1.5 mm axial reduction with rounded internal line angles, and a 1 mm chamfer finish line at the level of the gingival margin. With proper tissue management, a definitive impression was taken using light-bodied poly-vinyl siloxane (Imprint II Garant; 3M ESPE, St. Paul, MN, USA) and heavy-bodied poly-vinyl siloxane (Imprint II Garant; 3M ESPE, St. Paul, MN, USA). A working cast was poured with a type IV dental stone (Fujirock EP; GC, Leuven, Belgium).

### 2.2. Full-Contour Crown Fabrication

**Figure 1:**
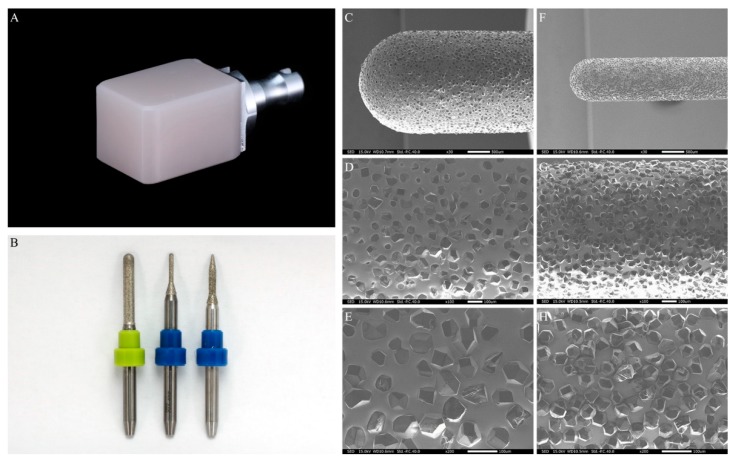
(**A**) A novel fully sintered niobium oxide containing yttria-stabilized tetragonal zirconia polycrystals ((Y,Nb)-TZP) block; (**B**) Novel milling tools used for the novel (Y,Nb)-TZP blocks: Φ2.5 mm (left), Φ1.0 mm (middle), and Φ0.5 mm (right); (**C**–**H**) SEM images for novel milling tools used for (Y,Nb)-TZP blocks: Φ2.5 mm with magnifications of ×30 (**C**), ×100 (**D**), ×200 (**E**) and Φ1.0 mm with magnifications of ×30 (**F**), ×100 (**G**), ×200 (**H**).

The working cast was scanned by dental laboratory scanner (D1000; 3Shape, Copenhagen, Denmark). A full-contour crown was designed by dental CAD software (Dental Designer; 3Shape, Copenhagen, Denmark). Parameters for restorations are as follows: cement gap 20 μm, extra cement gap 20 μm, distance to margin line 1.2 mm, smooth distance 0.3 mm, drill radius 0.5 mm, drill compensation offset 1.0 mm, margin line offset 50 μm, offset angle 70°, and extension offset 50 μm. Monolithic zirconia crowns were fabricated by two different fabrication processes and divided into two groups; (1) a chairside single-visit dentistry system (Chairside) group, involving a chairside 4-axis milling with a novel fully sintered (Y,Nb)-TZP block (ZirBlank-FS; Acucera, Kyunggi-do, Korea, Figure 1A and Table 1), and (2) a conventional laboratory system (Labside) group, involving 25%-oversized 5-axis milling with a partially sintered 3Y-TZP block (Luxen E2; DentalMax, Seoul, Korea) followed by a 12 h sintering process.

**Figure 2:**
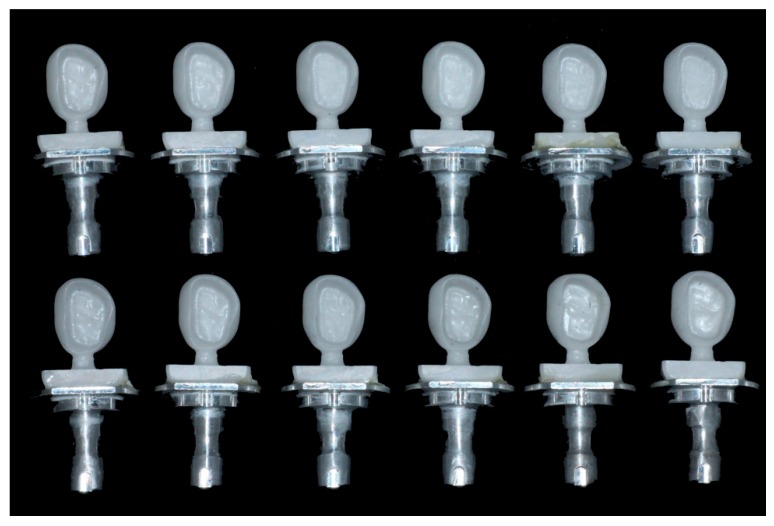
Monolithic zirconia crowns fabricated by milling fully sintered (Y,Nb)-TZP blocks in a chairside computer-aided design–computer-aided manufacturing (CAD–CAM) system for single-visit dentistry.

For the Chairside group (n = 12), milling was conducted with a dental CAM software (Hyper Dent; Follow-me! Technology, Munich, Germany) and a 4-axis wet milling machine (DWX-4W; Roland DG, Shizuoka, Japan) using novel diamond-milling burs (ED-BS; OSG, Aichi, Japan) designed for the novel (Y,Nb)-TZP block. Although equating the number of axes of each milling machine for the Chairside group and Labside group is desired, there was no available 5-axis chairside-milling machine and corresponding milling condition for milling a fully sintered (Y,Nb)-TZP block at the time we planned the experiment. Each set of milling tools used for fully sintered (Y,Nb)-TZP blocks consists of round-end blocky diamond grain-coated tungsten carbide burs of three different diameters (Φ2.5 mm, Φ1.0 mm, and Φ0.5 mm) (Figure 1B). Coated blocky diamond grain burs have superior wear resistance to the normally used regular diamond grain. SEM images for Φ2.5 mm and Φ1.0 mm milling tools are shown in Figure 1C–H. Using a tool set, 12 full-contour monolithic zirconia crowns were fabricated (Figure 2). No post-milling handling was conducted. The total fabrication time for each crown was recorded.

**Figure 3:**
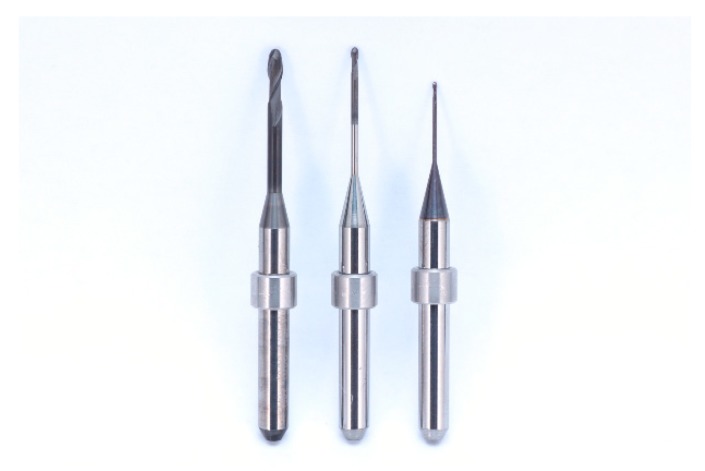
Milling tools used for partially sintered 3Y-TZP blocks: Φ2.0 mm (left), Φ1.0 mm (middle), and Φ0.6 mm (right).

For the Labside group, as a control group (n = 12), 12 zirconia crowns were milled using dental CAM software (Hyper Dent; Follow-me! Technology, Munich, Germany) and a 5-axis milling machine (Arum 5x-300; DoowonID, Daejeon, Korea) with milling burs (TZBE2000; KORLOY, Seoul, Korea). Each set of milling tools used for partially sintered 3Y-TZP blocks consists of ball-end diamond-coated tungsten carbide burs of three different diameters (Φ2.0 mm, Φ1.0 mm, and Φ0.6 mm) (Figure 3). After milling, full sintering was conducted with a sintering device (PDF-1000; DentalMax, Seoul, Korea) with sintering conditions as follows: starting temperature, 90 °C; firing temperature, 1530 °C; heating rate, 8.1 °C/min up to 1000 °C and 2.2 °C/min up to 1530 °C; cooling rate with 12.5 °C/min; and a total sintering time of 12 h. No post-sintering handling was conducted. The whole fabrication time for each crown was recorded.

### 2.3. Time Analysis

For the Chairside group, the fabrication time comprised the milling time only, whereas for the Labside group, the fabrication time comprised both the milling time and sintering time. For both groups, the total fabrication time for 12 crowns was recorded and the mean fabrication time for each crown was calculated by dividing the total fabrication time by the number of samples of each group.

### 2.4. Tool Wear Analysis

For the Chairside group, tool wear was assessed after every milling pass for the full-contour monolithic crown by measuring the diameters of the Φ2.5 mm roughening and Φ1.0 mm finishing burs with a digital caliper (Absolute Digimatic Caliper 500-182-50; Mitutoyo, Kanagawa, Japan). Because most of the milling was done with the Φ2.5 mm roughening bur and Φ1.0 mm finishing bur, the diameter of the Φ0.5 mm bur was not measured.

### 2.5. Trueness Analysis

The intaglio surfaces of all zirconia crowns were scanned with a highly accurate intraoral scanner (i500; Medit, Seoul, Korea) with an in vitro precision of 3.2 μm for a single tooth. With 3D inspection software (Geomagic Control X; Geomagic Inc, Morrisville, NC, USA), the scanned inner surface data was superimposed on the reference CAD data used for the fabrication of crowns. After setting the inner surface of the monolithic crown as an area of interest, initial alignment and best-fit alignment were conducted sequentially. The best-fit alignment uses an iterative closest point algorithm based on the point-to-point distance or linear distance measurement. The least-squares method (minimize the sums of squares of distances between the cloud of points) was used for the best-fit alignment. To compare the trueness of the inner surface of crowns, the RMS value between scan data and reference data was calculated by applying the following formula [23]:

(1) $$\mathrm{RMS}=\;\frac{\sqrt{\sum_{i=1}^{n}{\left(X_{1,i}-X_{2,i}\right)}^{2}}}{\sqrt{n}}$$

where *n* is the total number of measuring points, *X_1,i_* is the measuring point *i* on the reference data, and *X_2,i_* is the measuring point *i* on the crown scan data. The 3D color deviation maps were also obtained with maximum and minimum nominal values, or a tolerance level of 50 μm and maximum and minimum critical values of 500 μm. In accordance with Peters et al., who defined greater than 50 μm as poor trueness, nominal values or tolerance level was set to 50 μm, and deviations in the range of ± 50 μm are shown as green in the 3D color deviation map. Statistical software (IBM SPSS Statistics v21.0; IBM Corp., Armonk, NY, USA) was used for statistical analysis. The difference in trueness (RMS value) among consecutive monolithic crowns with each tool set was evaluated for both groups. The Jonckheere–Terpstra (J–T) test was applied to determine the influence of the number of consecutive milling processes on the RMS values of the inner surfaces of definitive crowns. For the test of normality, Kolmogorov–Smirnov and Shapiro–Wilk tests were performed. Levene’s test was used to assess the equality of variances. Independent samples t-test was used to determine the significant difference between the trueness of a full-contour monolithic crown fabricated with a chairside system and a laboratory system.

## 3. Results

### 3.1. Time Analysis

**Figure 4:**
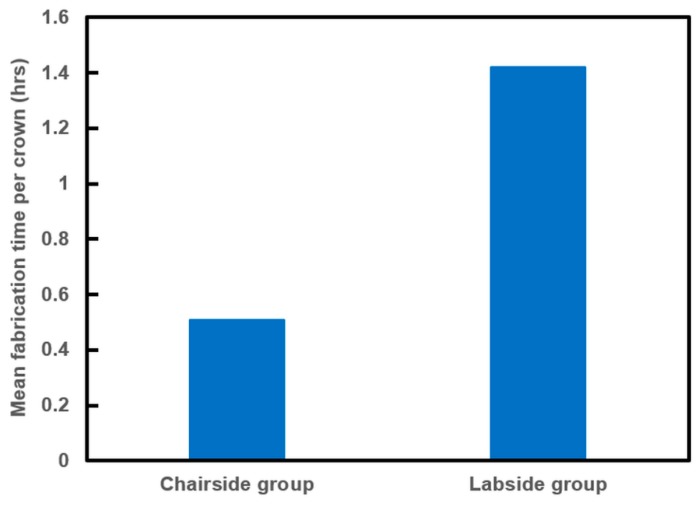
Mean fabrication time per crown in the chairside single-visit dentistry system (Chairside) group vs. the conventional laboratory system (Labside) group.

Fabrication time of 12 full-contour crowns was compared, and the results of the time analysis are shown in Figure 4 and Table 2. The mean fabrication time per crown was 0.52 h for the Chairside group and 1.42 h for the Labside group, respectively.

### 3.2. Tool Wear Analysis

**Figure 5:**
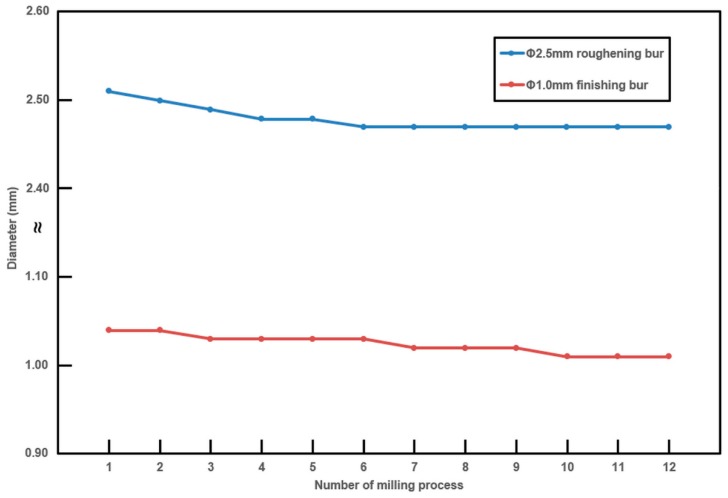
Changes in milling tool diameter for the chairside single-visit dentistry system (Chairside) group.

For the Chairside group, the diameter changes of the Φ2.5 mm roughening bur and Φ1.0 mm finishing bur after every milling are shown in Figure 5. A decrease in diameter was observed for both burs. Compared with the first measurement, the 12^th^ measurement was decreased by 2% for the Φ2.5 mm bur, and 5% for the Φ1.0 mm milling bur.

### 3.3. Trueness Analysis

**Figure 6:**
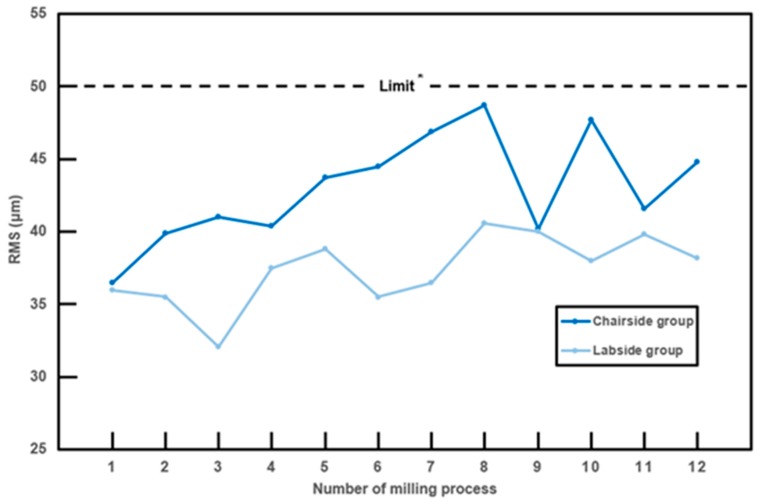
Changes in root mean square (RMS) value in the chairside single-visit dentistry system (Chairside) group vs. the conventional laboratory system (Labside) group. *Clinically acceptable limit proposed by Peters et al. [18].

**Figure 7:**
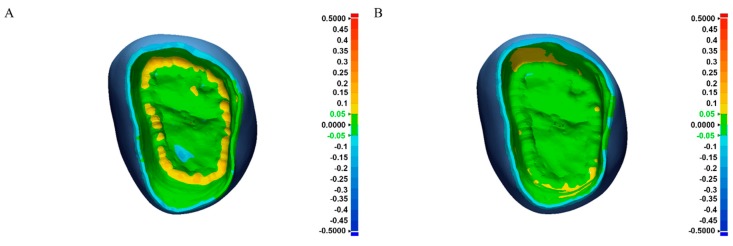
3D color deviation maps for the chairside single-visit dentistry system (Chairside) group (**A**) and conventional laboratory system (Labside) group (**B**). Positive deviation was displayed with yellow to red colors and negative deviation with cyan to blue.

The RMS values of the inner surfaces of 12 full-contour crowns in both groups significantly changed as the number of milling processes continued (p = 0.02, standard J–T value = 2.331 for the Chairside group and p = 0.046; standard J–T value = 1.993 for the Labside group by J–T test). There was an increasing tendency of RMS values as milling cycles proceeded (Figure 6). All RMS values of both the Chairside and Labside groups were less than 50 μm in this study, even with tool wear. The means and standard deviations of RMS values of the inner surfaces of monolithic zirconia crowns were tested, and the results of the independent t-test are shown in Table 3. The mean RMS value was 43.0 ± 3.67 μm for the Chairside group and 37.4 ± 2.41 μm for the Labside group. Both sets of data passed the tests of equality of variance. All RMS values of both the Chairside and Labside group were less than 50 μm, but the RMS values of the Chairside group were significantly greater than the Labside group (P < 0.05). The color deviation map represents the 3D surface deviation between the scan data of the inner surface of monolithic zirconia crowns and reference CAD data (Figure 7). The yellow to red areas represent positive deviation, which means that the surface of the scan data is higher than the reference data. The cyan to blue areas represent negative deviation, which is the opposite to positive deviation. The green area represents surface deviation within the acceptable limit. Most inner surfaces and marginal areas of both groups showed surface deviations within the acceptable limit of ± 50 μm (Figure 7). However, in the area near the occlusoaxial line angle, the Chairside group exhibited a small band-like yellow region, suggesting a positive surface deviation of over 50 μm.

## 4. Discussion

Based on the results of this study, the mean RMS values were 43.0 ± 3.67 μm for the Chairside group and 37.4 ± 2.41 μm for the Labside group. Even though the Chairside group showed a significantly greater RMS value than the Labside group, all RMS values of both the Chairside and Labside groups were less than 50 μm and were in the clinically acceptable range [18]. Some studies deal with the trueness of the inner or intaglio surfaces of crowns. In a recent study, zirconia crowns fabricated by milling partially sintered zirconia blocks using a five-axis milling machine showed an RMS value of 43 ± 12 μm [5]. There have been no studies comparing the internal trueness of CAD–CAM zirconia crowns fabricated from fully sintered and partially sintered zirconia blocks. For glass-ceramic crowns, RMS values ranged from 31.4 μm to 55.0 μm for 4-axis milling machines, and from 37.3 μm to 37.6 μm for 5-axis milling machines [13]. For crowns fabricated by milling a polyurethane block, the RMS value was 16 ± 7 μm with three burs [15]. For crowns fabricated by milling polymethyl methacrylate, RMS values were 14.6 ± 1.2 μm [24]. Our study shows results that are consistent with other studies, which have demonstrated clinically acceptable trueness. Although Peters et al. proposed that an RMS value of less than 10 μm is an excellent fit [18], no study has shown experimental RMS values less than 10 μm. Moreover, the proposal of Peters et al. is not evidence-based [18]; thus, new guidelines for determining good or excellent trueness should be established. 

In this study, we used a four-axis chairside-milling machine for the Chairside group and five-axis laboratory-milling system for the Labside group. At the time we conducted the experiment, there was no available five-axis chairside-milling system for milling fully sintered zirconia blocks. Importantly, this study aimed to compare the outcomes of two different CAD–CAM systems (a chairside single-visit dentistry system and conventional laboratory system) but not the outcomes of two different zirconia blocks (fully sintered zirconia block and partially sintered zirconia block). For the chairside-milling machine, 3- or 4-axis machines are widely used; whereas, for the laboratory-milling machine, 5-axis machines are more widely used [13]. Compared with 3-axis or 4-axis milling machines, a 5-axis milling machine has greater accuracy but is more expensive [25]. Therefore, for the laboratory-milling system in this study, we determined that instead of a four-axis laboratory-milling system, a five-axis laboratory-milling system, which is currently used in dental laboratories to fabricate zirconia crown, was most appropriate. Our finding of lower trueness in the Chairside group could be attributed to the fewer number of axes of the milling machine. Nevertheless, the chairside system showed clinically acceptable results. Future studies are desirable to repeat this experiment with milling machines of equal axes for a better comparison. 

The mean fabrication time for 12 crowns was about 0.52 h for a fully sintered (Y,Nb)-TZP block in the chairside system and about 1.42 h for a partially sintered 3Y-TZP block in the laboratory system. The difference in mean fabrication time will rapidly increase by manufacturing only one crown for each fabrication system. The fabrication of a crown from a commercially widely available partially sintered 3Y-TZP block takes more than 12 h, including the milling and sintering processes. Although speed or high-speed sintering processes greatly reduce the sintering time, it still takes too long and is thus not suitable for a single-visit chairside restoration [2,3]. Also, the questionable properties of zirconia fabricated by a speed or high-speed sintering process have limitations in clinical application [2,3]. Therefore, regarding the fabrication time, milling a fully sintered (Y,Nb)-TZP block in the chairside system has great advantages for single-visit chairside restoration that cannot be achieved using the laboratory system. Overall, the chairside system in this study showed rapidly fabricated crowns with clinically acceptable trueness, even with a four-axis milling machine, and may be a promising candidate for a single-visit restoration system. One advantage of a single-visit restoration system is the decrease of time consumed by patients or individuals. This time includes travelling to the dental office, waiting in the dental office, and travelling home. It is not only the private costs, but also the social costs. In addition, by decreasing the time, it is more convenient for the patient to receive the dental service. Thus, the chairside system in this study might contribute to decrease both private and social costs and increase the patient convenience.

According to the 3D color deviation maps, compared with the Labside group, the Chairside group exhibited more positive deviation values, especially at the area near the occlusoaxial line angles. Positive deviation values represent larger crowns than the reference and thus suggest the presence of undermilling. This undermilling might be due to the limitations of tools for milling hard fully sintered (Y,Nb)-TZP blocks in deep areas. Some studies have revealed concerns in thin areas when milling a fully sintered zirconia block [6,7]. Thus, our concerns in the deep areas when milling a fully sintered block add to the concerns about thin areas. Milling parameters for optimizing milling a fully sintered zirconia block should be developed in future studies.

In this study, we 3D analyzed the trueness of the inner surfaces of full-contoured crowns in the Chairside group and Labside group. No studies have compared fully sintered milling and partially sintered milling with respect to trueness. Actually, trueness is not a measure of fit. A few articles have compared the marginal or internal fit of zirconia crowns from fully sintered zirconia blocks and partially sintered zirconia blocks, showing that fully sintered milling usually results in a better fit than a partially sintered milling in terms of marginal and internal fit [6]. Measuring the marginal or internal gap in two dimensions (2D), such as the widely used silicon replica technique [26,27], in practical cases limits the number of measurement points, and has low reproducibility and distance in 2D [28,29]. Ideally, the gap or discrepancy should be measured in 3D using a normal vector or projection at each measuring point. Trueness is a 3D measurement used to calculate the distance in 3D at measurement points by a computer and covers a lot of randomly distributed measurement points over the interest region. Thus, 3D trueness analysis is more credible. It might be interesting to compare 3D trueness and values of fit in 2D from various techniques, i.e., silicon replica technique or micro CT technique. Few studies, including this study, have compared fully sintered milling and partially sintered milling with respect to trueness or fit. Thus, more studies with 3D or 2D analysis are needed to compare the outcomes of fully sintered and partially sintered zirconia blocks.

In this study, we used commercial 3D inspection software, Geomagic Control X, which usually adopts methodology based on the point-to-point distance or linear point measurement. 3D inspection result such as trueness is affected by not only the accuracy of scanners, but also the methodology for data analysis. In recent study, a point-to-surface distance measurement or volumetric error approach using a custom algorithm has been suggested for better reliability of the inspection procedure [30]. Better methodology for reliable analysis should be developed in further studies.

Tool wear was assessed for the Chairside group because of the concern of robust milling. Since there are no established criteria for the assessment of tool wear, we planned to measure the milling tool diameter to evaluate tool wear, considering that tool wear and the loss of tool mass might take place due to framework wear and the loss of diamond grit [22]. A decrease in diameter was observed for both burs. Although tool wear could affect the trueness of crowns for the Chairside group and increasing tendency of RMS values from the J–T test was observed for both groups, all the RMS values were less than 50 μm which were clinically acceptable for both groups and pooling of RMS values of crowns for both groups was conducted. In addition, the dental technician does not change the milling tool during milling of several tens of crowns in the laboratory system, owing to clinically acceptable outcomes and cost-effectiveness. Indeed, there is no definitive guideline from the manufacturer about when to change milling tool, such as before or after tool breakdown, depending on the material–tool combination. From this study, the decrease in diameter of the milling tool and increasing tendency of RMS values from the J–T test do not directly indicate the need for changing milling burs. Further studies about milling tool wear are needed to evaluate tool survival regarding not only tool breakdown, diameter, mass, or surface roughness but also the outcomes of restoration.

For a fully sintered zirconia block, lack of a post-milling sintering process is advantageous in terms of fabrication time, while it is, at the same time, disadvantageous in terms of surface flaws, surface roughness and phase transformation. Even though the Chairside group showed clinically acceptable trueness in this study, the possibility of presence of surface flaws, an increased surface roughness and increased monoclinic phase fraction due to robust milling of a fully sintered zirconia block, might lead to concerns about the reliability and longevity of prosthesis [9,10,11,12]. Some studies suggest that the problem, if it happens, can be solved by polishing, regeneration firing or glazing [9,31]. Nevertheless, this study did not focus on these topics, thus, further study should be conducted covering these topics.

There are some limitations in this study. Increased sample sizes in each milling cycle for both groups are desired for further statistical analysis. Further, we only focused on the inner surface of the crown; thus, the lower RMS values for the inner surface do not necessarily reflect the lower RMS values for the outer surface. Performance of crowns, such as fracture strength or long-term mechanical properties, were not investigated in this study and further study covering such a performance of crowns is needed.

## 5. Conclusions

Within the limitations of this in vitro study, the following conclusions were drawn:

1. Single-visit zirconia crown restoration that meets the clinical trueness requirement can be realized via milling a fully sintered (Y,Nb)-TZP block with a chairside CAD–CAM system.
2. The trueness outcomes of both Chairside and Labside groups were clinically acceptable, although significantly lower trueness was observed in the Chairside group than in the Labside group.
3. The mean fabrication time was 0.52 h for the Chairside group and 1.42 h for the Labside group.
4. For the Chairside group, the trueness outcomes of the crowns were all clinically acceptable, even though the diameter of the milling tools decreased.

## Figures and Tables

**Table 1:** Mechanical and optical properties of a novel fully sintered (Y,Nb)-TZP block for a chairside single-visit dentistry system (Chairside) group.

Time	Chairside Group
Flexural strength (MPa)	760
Toughness (MPa·m^1/2^)	7.4
Hardness (GPa)	8.6
Machinability (m^1/2^ × 10^3^) (defined as the ratio of fracture toughness/hardness)	0.86
Transmittance (%)	58

**Table 2:** Fabrication time of 12 monolithic crowns in the chairside single-visit dentistry system (Chairside) group and conventional laboratory system (Labside) group.

Time	Chairside Group	Labside Group
Milling time (h)	6.2	5.0
Sintering time (h)	-	12.0
Total fabrication time (h)	6.2	17.0
Mean fabrication time per crown (h)	0.52	1.42

**Table 3:** Trueness (RMS value) of the inner surfaces of the monolithic crowns of the chairside single-visit dentistry system (Chairside) group and conventional laboratory system (Labside) group.

Area	Chairside Group (μm)		Labside Group (μm)		p
	RMS ± SD	95% CI	RMS ± SD	95% CI	
Inner	43.0 ± 3.67	40.7–45.3	37.4 ± 2.41	35.8–38.9	<0.001

CI, confidence interval; RMS, root mean square; SD, standard deviation.
